# NPR1 suppresses *Candidatus* Liberibacter asiaticus-induced callose and reactive oxygen species accumulation

**DOI:** 10.1093/plphys/kiaf532

**Published:** 2025-10-21

**Authors:** Poulami Sarkar, Choaa El-Mohtar, Chunxia Wang, Donielle Turner, Stacy Welker, Cecile J Robertson, Vladimir Orbović, Zhonglin Mou, Amit Levy

**Affiliations:** Citrus Research and Education Center, Lake Alfred, University of Florida, Lake Alfred, FL 33850, USA; Citrus Research and Education Center, Lake Alfred, University of Florida, Lake Alfred, FL 33850, USA; Department of Plant Pathology, University of Florida, Gainesville, FL 32602, USA; Citrus Research and Education Center, Lake Alfred, University of Florida, Lake Alfred, FL 33850, USA; Citrus Research and Education Center, Lake Alfred, University of Florida, Lake Alfred, FL 33850, USA; Citrus Research and Education Center, Lake Alfred, University of Florida, Lake Alfred, FL 33850, USA; Citrus Research and Education Center, Lake Alfred, University of Florida, Lake Alfred, FL 33850, USA; Citrus Research and Education Center, Lake Alfred, University of Florida, Lake Alfred, FL 33850, USA; Department of Microbiology and Cell Sciences, University of Florida, Gainesville, FL 32603, USA; Citrus Research and Education Center, Lake Alfred, University of Florida, Lake Alfred, FL 33850, USA; Department of Plant Pathology, University of Florida, Gainesville, FL 32602, USA

## Abstract

Huanglongbing (HLB), a devastating citrus disease caused by *Candidatus* Liberibacter asiaticus (*C*Las), triggers persistent immune activation marked by excessive callose deposition and reactive oxygen species (ROS) accumulation, which impairs phloem function. This maladaptive response has led to HLB being described as a “pathogen-triggered immune disease”. Overexpression of the Arabidopsis (*Arabidopsis thaliana*) *NONEXPRESSOR OF PATHOGENESIS-RELATED GENES1* (*AtNPR1*) gene, a master regulator of systemic acquired resistance (SAR), confers robust HLB tolerance in susceptible citrus varieties, with transgenic lines exhibiting minimal or no disease symptoms following *C*Las infection. However, the mechanism underlying this tolerance remains unclear. In this study, we demonstrate that *AtNPR1* restores immune homeostasis in *C*Las-infected trees by suppressing callose and ROS hyperaccumulation, thereby alleviating HLB symptom development. Similarly, silencing the *Citrus sinensis* homolog of *NPR3* (*CsNPR3*), a negative regulator of SAR, mitigates *C*Las-induced immune overactivation and enhances HLB tolerance. Notably, salicylic acid (SA) levels are lower in *AtNPR1*-overexpressing citrus plants than wild-type controls after *C*Las infection, consistent with NPR1's role in negative feedback regulation of pathogen-induced SA accumulation. In *Arabidopsis*, overexpression of *AtNPR1* or disruption of *AtNPR3*/*AtNPR4* also attenuates pathogen-induced callose and ROS responses. Together, these findings reveal conserved roles for NPR1/NPR3/NPR4 in immune regulation across species and suggest that HLB susceptibility in commercial citrus varieties stems from a diminished capacity to maintain immune balance.

## Teaser

Expression of the key immune regulator AtNPR1 in citrus generates tolerance to HLB disease by preventing excess callose and ROS accumulation and maintaining immune balance.

## Introduction

Huanglongbing (HLB) or citrus greening, caused by the bacterial pathogen *Candidatus* Liberibacter asiaticus (*C*Las), is a devastating disease that afflicts citrus trees globally, leading to substantial production costs and declines in fruit quality. *C*Las infection triggers robust phloem immune responses characterized by excessive accumulation of reactive oxygen species (ROS) and increased deposition of callose. While a basal level of callose is transiently present in healthy sieve plates, callose can rapidly accumulate and block the sieve pores upon pathogen attack (Wang et al. 2021). ROS are also naturally produced during normal plant growth; however, their accumulation becomes excessive following *C*Las infection, leading to oxidative cell death (Bailly et al. 2008; Ye et al. 2021). These responses trigger phloem collapse, which obstructs the movement of photoassimilates from source leaves to sink tissues (Kim et al. 2009; Achor et al. 2010; Koh et al. 2012; Ma et al. 2022; Welker et al. 2022). This obstruction is believed to cause the development of HLB symptoms, including stunted growth, yellow and blotchy leaves, and lopsided fruits with aborted seeds (Schneider 1968; da Graça et al. 2016). Current strategies to manage HLB symptom development and its spread mostly rely on foliar application and trunk injection of antimicrobials, with limited effectiveness and unknown physiological effects on the trees (Zhang et al. 2014; Young et al. 2017; Archer and Albrecht 2023).

Comparative studies of HLB-susceptible and tolerant citrus varieties have uncovered a potential role of immune responses, particularly systemic acquired resistance (SAR), in mediating HLB tolerance (Wang et al. 2016; Weber et al. 2022). SAR is a long-lasting, broad-spectrum immune response induced by mobile signals produced at the primary infection site and requires the phytohormone salicylic acid (SA) (Gaffney et al. 1993; Fu and Dong 2013). A key player in SA-mediated signaling is NONEXPRESSOR OF PATHOGENESIS-RELATED GENES1 (NPR1), which acts as an SA receptor (Cao et al. 1998; Wu et al. 2012). Upon SA-induced redox changes, NPR1 moves into the nucleus to interact with TGA transcription factors to activate the transcription of defense genes, including *PR* genes (Zhang et al. 1999; Després et al. 2000, 2003; Kinkema et al. 2000; Fan and Dong 2002; Kim and Delaney 2002; Mou et al. 2003; Kumar et al. 2022). The NPR1 paralogs, NPR3 and NPR4, are also SA receptors (Fu et al. 2012), which play opposite roles in regulating SA-induced transcriptional changes (Ding et al. 2018). Interestingly, upon *C*Las infection, 4 *NPR1*-like genes were significantly upregulated in HLB-tolerant “Jackson” grapefruit-like-hybrid trees and only one was upregulated in the closely related susceptible “Marsh” grapefruit trees (Wang et al. 2016). Moreover, *NPR1*-like genes were not induced by *C*Las infection in susceptible “Valencia” sweet orange (Martinelli et al. 2012). Although exogenous application of SA showed improved tolerance to HLB, the effects were transient (Mann et al. 2011).

In citrus, the importance of NPR1-mediated signaling in generating disease tolerance has been underscored by multiple transgenic studies. Overexpression of *Arabidopsis thaliana NPR1* (*AtNPR1*) or its citrus ortholog *CtNH1* (*CITRUS NPR1 HOMOLOG 1*) in “Hamlin” sweet orange and/or “Duncan” grapefruit was initially found to enhance resistance to citrus canker (Zhang et al. 2010; Chen et al. 2013; Wu et al. 2021). Subsequent intensive greenhouse screening identified independent transgenic lines that accumulate high levels of the AtNPR1 protein and only occasionally displayed mild HLB symptoms, and the progeny plants of these transgenic lines retained the same levels of HLB tolerance (Robertson et al. 2018). Independently produced transgenic “Hamlin” and “Valencia” sweet orange expressing *AtNPR1* also exhibited reduced HLB disease severity under high disease pressure in the field (Dutt et al. 2015). Although overexpression of *AtNPR1* in citrus has been shown to elevate the expression of a group of defense-related genes, including *PATHOGENESIS-RELATED PROTEIN 1* (*PR1*) (Dutt et al. 2015; Qiu et al. 2020), the mechanisms preventing HLB disease symptom development remain unclear. Additionally, overexpression of the *AtNPR1* gene or knocking out the potato *NPR3* ortholog in microbial hairy roots and in potato significantly decreased the titers of *Candidatus* Liberibacter solanacearum, which causes potato zebra chip, a disease similar to HLB (Irigoyen et al. 2020; Ramasamy et al. 2024). These results suggest a potential correlation between NPR1/NPR3-mediated signaling and HLB tolerance in citrus.

HLB has recently been described as an immune disease where excessive and prolonged immune responses lead to cell death in the phloem, driven by ROS accumulation (Ma et al. 2022). This understanding, however, appears to conflict with findings that overexpression of *AtNPR1* results in HLB tolerance (Dutt et al. 2015; Robertson et al. 2018). In this study, we characterized *C*Las-induced callose deposition and ROS accumulation in transgenic “Hamlin” sweet orange and “Duncan” grapefruit trees overexpressing the *AtNPR1* gene (hereafter *AtNPR1-OE*). Additionally, we explored the role of *Citrus sinensis NPR3* (*CsNPR3*), the closest citrus homolog of *AtNPR3* and *AtNPR4*, which encode negative immune regulators (Zhang et al. 2006), in HLB via *Citrus tristeza virus*-based RNA interference (CTV-RNAi) (Hajeri et al. 2014). Subsequently, we investigated the roles of *AtNPR1* and *AtNPR3*/*AtNPR4* in regulating callose and ROS accumulation in *Arabidopsis* induced by the bacterial pathogen *Pseudomonas syringae* pv. *maculicola* ES4326 (*Psm*). Our findings revealed conserved functions of *NPR1/NPR3/NPR4* in regulating plant immune responses in both citrus and *Arabidopsis*.

## Results

### Callose deposition occurs early in citrus and precedes ROS accumulation in response to *C*Las infection

**Figure 1.**
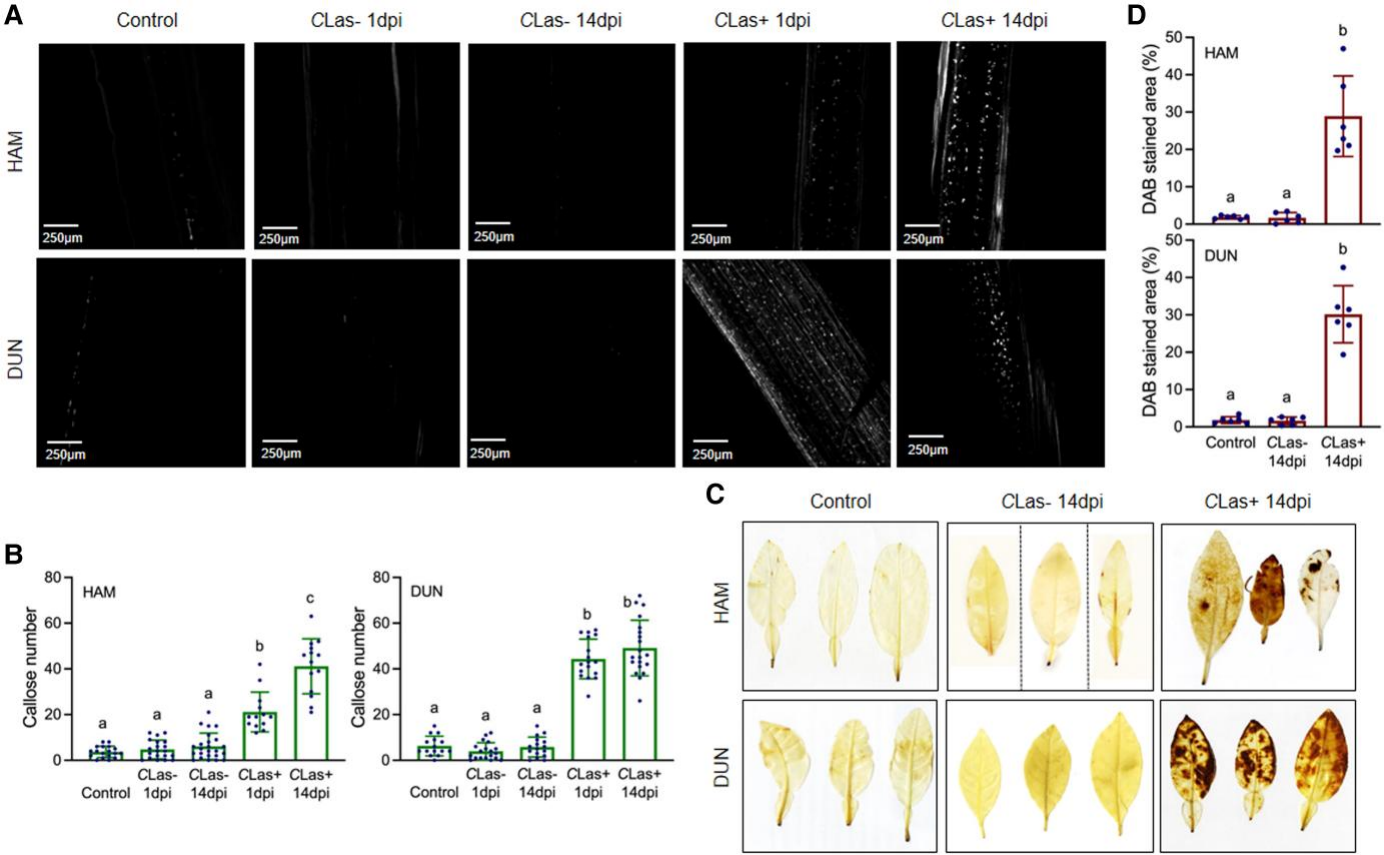
*C*Las-induced callose deposition and ROS accumulation. **A)** Callose deposition revealed by aniline blue staining in citrus stems adjacent to the leaves inoculated with *C*Las-free (*C*Las−) or *C*Las-infected (*C*Las+) psyllids at 1 and 14 dpi. Representative images are shown. Control, uninoculated healthy stems; HAM, Hamlin; DUN, Duncan. **B)** Number of callose depositions in the control and the citrus stems adjacent to the leaves inoculated with *C*Las-free or *C*Las-infected psyllids at 1 and 14 dpi. Bars represent “means ± standard deviation (Sd)” (*n* = 13 to 24). Data from 3 independent experiments were combined. Different letters above the bars denote significant differences (*P* < 0.05; one-way ANOVA with Tukey's test). **C)** ROS accumulation revealed by DAB staining in citrus leaves inoculated with *C*Las-free or *C*Las-infected psyllids at 14 dpi. Representative images are shown. Control: uninoculated healthy citrus leaves. Scale bars represent 1 cm. **D)** Percentages of leaf areas stained with DAB in the control and the citrus leaves inoculated with *C*Las-free or *C*Las-infected psyllids at 14 dpi. Bars represent “means ± Sd” (*n* = 6). Different letters above the bars denote significant differences (*P* < 0.05; one-way ANOVA with Tukey's test).

To understand the effects of citrus immunity on HLB tolerance, we aimed to establish an early timeframe for callose and ROS accumulation in citrus in response to *C*Las. Overactivation of citrus immune responses is believed to stimulate HLB symptoms because of callose and ROS accumulation (Ma et al. 2022). It has been shown that H_2_O_2_ and callose accumulated at significantly higher levels in young flushes produced by *C*Las-positive citrus plants than in those on healthy plants at 15 and 18 day post-bud initiation, respectively, indicating that *C*Las induces ROS production earlier than callose deposition in new flushes on *C*Las-positive plants (Ma et al. 2022). To determine the kinetics of *C*Las-induced callose and ROS accumulation in naïve healthy citrus plants, we inoculated “Hamlin” sweet orange and “Duncan” grapefruit with *C*Las-free and *C*Las-infected psyllids and analyzed callose deposition (Supplementary Fig. S1) and ROS accumulation at 1 and 14 day post-inoculation (dpi). Surprisingly, significant induction of callose deposition occurred within 1 dpi with *C*Las-infected psyllids, and callose levels further increased at 14 dpi (Fig. 1, A and B). In contrast, inoculation with *C*Las-free psyllids did not increase callose levels in the phloem of either the “Hamlin” or “Duncan” plants (Fig. 1, A and B). Moreover, significant ROS accumulation was detected at 14 dpi with *C*Las-infected psyllids (Supplementary Fig. S2; Fig. 1, C and D). This prompted us to designate 14 dpi as a critical time point for further experiments in citrus. Inoculation with *C*Las-free psyllids did not cause significant ROS generation compared with the uninoculated controls. Taken together, these results indicate that *C*Las-triggered callose deposition happens considerably earlier (at 1 dpi) than previously thought and is followed by ROS accumulation.

### Overexpression of *AtNPR1* in citrus increases basal callose levels and suppresses *C*Las-induced callose deposition and ROS accumulation

**Figure 2.**
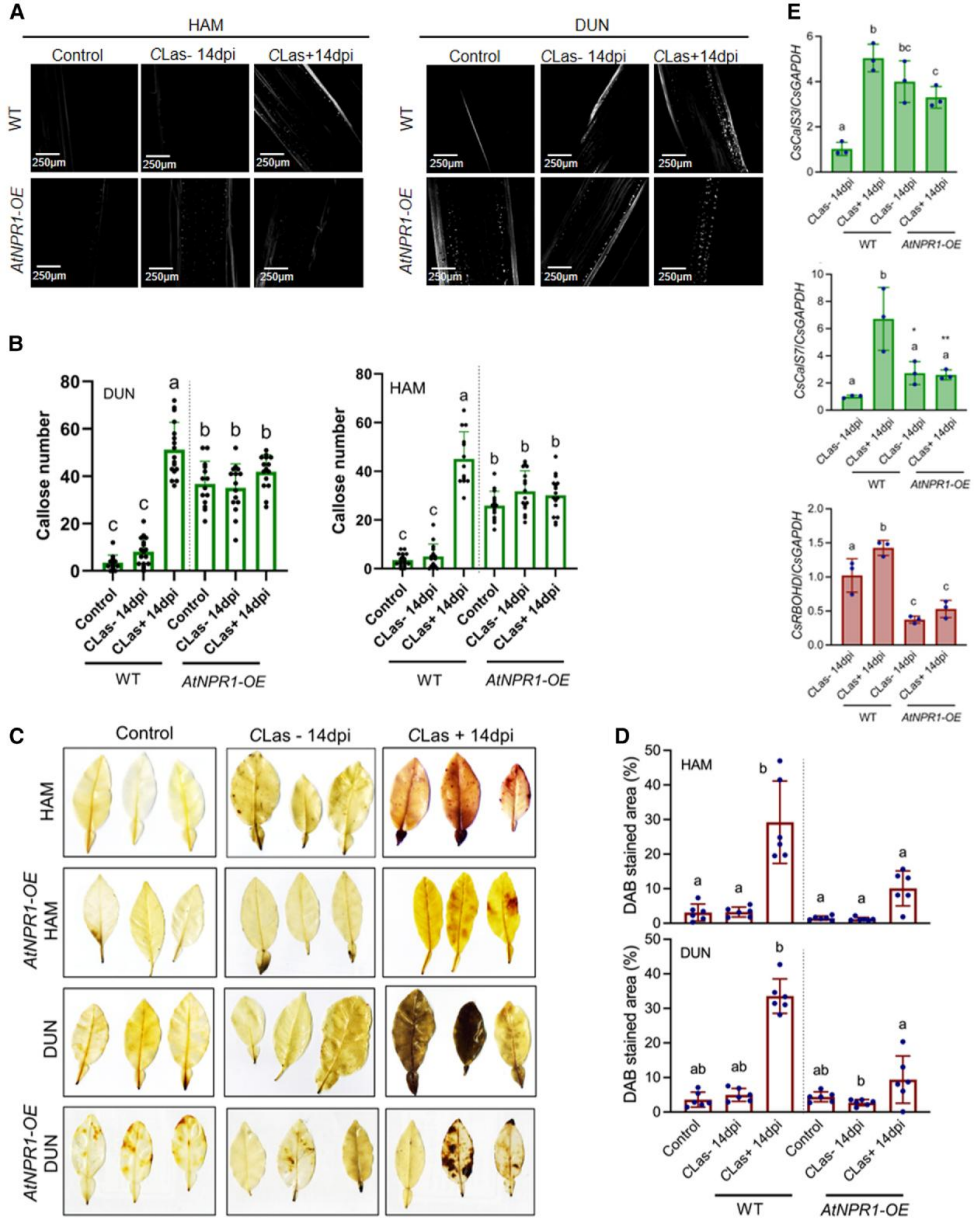
Callose deposition and ROS accumuation in citrus *AtNPR1-OE* plants. **A)** Callose deposition revealed by aniline blue staining in WT and *AtNPR1-OE* citrus stems adjacent to the leaves inoculated with *C*Las-free (*C*Las−) or *C*Las-infected (*C*Las+) psyllids at 14 dpi. **B)** Number of callose depositions in the WT and *AtNPR1-OE* citrus stems adjacent to the leaves inoculated with *C*Las-free or *C*Las-infected psyllids at 14 dpi. Bars represent “means ± Sd” (*n* = 12 to 18). Data from 3 independent experiments were combined. Different letters above the bars denote significant differences (*P* < 0.05; one-way ANOVA with Tukey's test). **C)** ROS accumulation revealed by DAB staining in WT and *AtNPR1-OE* citrus leaves inoculated with *C*Las-free or *C*Las-infected psyllids at 14 dpi. Scale bars represent 1 cm. (Images in the box are photographed at the same time). **D)** Percentages of leaf areas stained with DAB in the WT and *AtNPR1-OE* citrus leaves inoculated with *C*Las-free or *C*Las-infected psyllids at 14 dpi. Bars represent “means ± Sd” (*n* = 6). Different letters above the bars denote significant differences (*P* < 0.05; one-way ANOVA with Tukey's test). **E)** Expression of *CsCalS3*, *CsCalS7*, and *CsRBOHD* in WT and *AtNPR1-OE* “Duncan” grapefruit leaves inoculated with *C*Las-free (*C*Las−) or *C*Las-infected (*C*Las+) psyllids at 14 dpi. Bars represent “means ± Sd” (*n* = 3). Different letters above the bars denote significant differences (*P* < 0.05; one-way ANOVA with Tukey's test). Asterisks indicate having significant difference from the WT inoculated with *C*Las-free psyllids (*P < 0.05; **P < 0.01: Student's *t*-test).

NPR1 is known to negatively regulate pathogen-triggered ROS accumulation, but to positively contribute to pathogen-induced callose deposition (Dong et al. 2008; Chen et al. 2017; Seo et al. 2020). To understand how *AtNPR1*-mediated HLB tolerance can be correlated with pathogen-induced callose deposition and ROS production, we opted to investigate the effect of *AtNPR1* on callose and ROS regulation in citrus. We inoculated *AtNPR1-OE* and non-transgenic (hereafter wild-type [WT]) “Hamlin” and “Duncan” plants with either *C*Las-free or *C*Las-infected psyllids and examined callose and ROS accumulation at 14 dpi. As shown in Fig. 2, *C*Las-free psyllids did not induce callose deposition and ROS accumulation, whereas *C*Las-infected psyllids induced heavy deposition of callose and significant accumulation of ROS in the WT citrus plants. Surprisingly, the *AtNPR1-OE* citrus plants exhibited increased basal callose levels in the phloem tissues when uninoculated, and inoculation with either *C*Las-free or *C*Las-infected psyllids did not significantly increase the callose levels (Fig. 2, A and B). The callose levels in the *AtNPR1-OE* citrus plants are significantly higher than those in the WT when inoculated with *C*Las-free psyllids but are significantly lower than those in the WT when inoculated with *C*Las-infected psyllids (Fig. 2, A and B). Moreover, the *AtNPR1-OE* citrus plants showed slightly increased ROS levels after inoculation with *C*Las-infected psyllids compared with inoculation with *C*Las-free psyllids or without inoculation (Fig. 2, C and D). The ROS levels in the *AtNPR1-OE* citrus plants were much lower than those in the WT citrus plants after inoculation with *C*Las-infected psyllids (Fig. 2, C and D).

To ascertain the elevated basal levels of callose and the reduced induction of *C*Las-mediated callose deposition and ROS accumulation in the *AtNPR1-OE* citrus plants, we examined the expression levels of *C. sinensis CALLOSE SYNTHASE 3* and 7 (*CsCalS3*and *CsCalS7*, respectively) as well as *RESPIRATORY BURST OXIDASE HOMOLOG D* (*CsRBOHD*) in *AtNPR1-OE* and WT “Duncan” plants with or without *C*Las infection. As shown in Fig. 2E, the basal transcript levels of *CsCalS3* and *CsCalS7* are significantly higher in the *AtNPR1*-OE “Duncan” plants than in the WT. However, at 14 dpi with *C*Las-infected psyllids, *CsCalS3* and *CsCalS7* transcripts were significantly upregulated in the WT “Duncan” but were not induced in the *AtNPR1*-OE “Duncan” plants (Fig. 2E). On the other hand, the basal expression level of *CsRBOHD* was significantly lower in the *AtNPR1*-OE plants than in the WT (Fig. 2E). Inoculation with *C*Las-infected psyllids significantly upregulated *CsRBOHD* transcription in the WT but did not affect *CsRBOHD* expression in the *AtNPR1*-OE “Duncan” plants (Fig. 2E). These results suggest that *AtNPR1* may modulate callose deposition and ROS accumulation in citrus by regulating the expression of *CsCalS3/7* and *CsRBOHD*, respectively.

### *C*Las-induced vascular tissue alterations are minimized in HLB-infected *AtNPR1-OE* citrus

**Figure 3.**
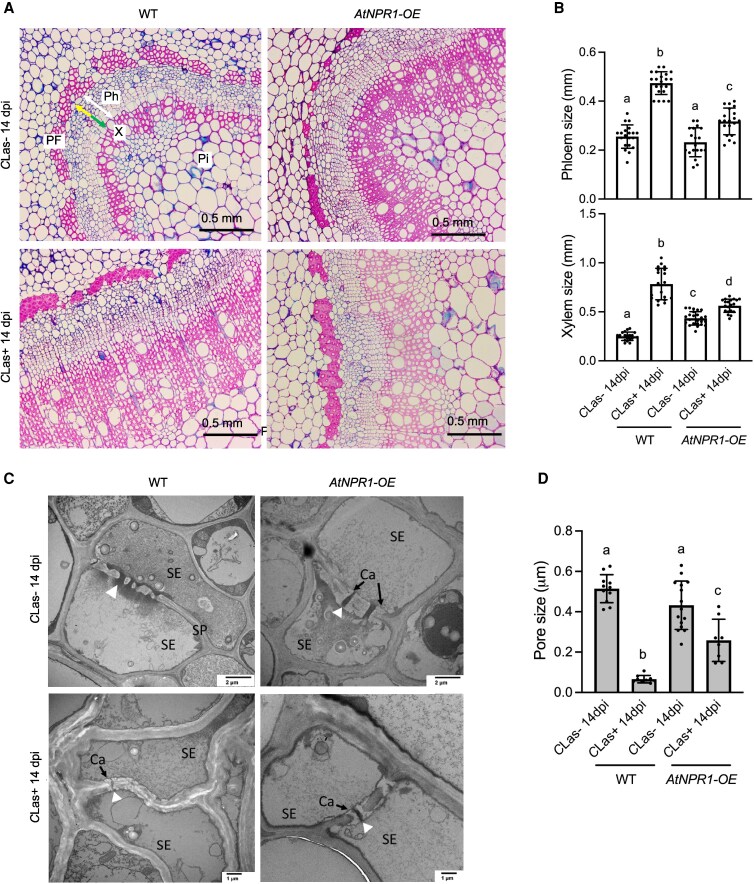
*C*Las-induced vascular tissue alterations and sieve pore plugging in citrus *AtNPR1-OE* plants. **A)** Light microscopy images of methylene blue- and basic fuchsin-stained stem sections of WT and *AtNPR1-OE* “Duncan” grapefruit plants inoculated with *C*Las-free (*C*Las−) or *C*Las-infected (*C*Las+) psyllids at 14 dpi. PF, phloem-fibers; Ph, phloem; X, xylem; Pi, pith; white arrow: phloem size; green arrow: new phloem; yellow arrow: replacement phloem. **B)** The phloem and xylem sizes in the WT and *AtNPR1-OE* “Duncan” grapefruit plants inoculated with *C*Las-free or *C*Las-infected psyllids at 14 dpi. Bars represent “means ± Sd” (*n* = 19 to 24). Different letters above the bars denote significant differences (*P* < 0.05; one-way ANOVA with Tukey's test). **C)** TEM images of the WT and *AtNPR1-OE* “Duncan” grapefruit plants inoculated with *C*Las-free or *C*Las-infected psyllids at 14 dpi. Ca, callose; SP, sieve plate; SE, sieve element; white arrowhead: open passage between 2 sieve elements. **D)** Pore sizes between sieve elements in the WT and *AtNPR1-OE* “Duncan” grapefruit plants inoculated with *C*Las-free or *C*Las-infected psyllids at 14 dpi. Bars represent “means ± Sd” (*n* = 8 to 15). Different letters above the bars denote significant differences (*P* < 0.05; one-way ANOVA with Tukey's test).

*C*Las infection causes vascular tissue alterations and sieve pore plugging in susceptible citrus cultivars, resulting in HLB symptom development (Brodersen et al. 2014; Deng et al. 2019; Achor et al. 2020). To investigate the difference in the vascular bundles between *AtNPR1-OE* and WT citrus plants, we carried out thin-section light and transmission electron microscopy (TEM) analysis of the vasculature following inoculation with *C*Las-free or *C*Las-infected psyllids. As shown in Fig. 3, A and B, the *AtNPR1-OE* and WT “Duncan” plants displayed similar phloem size, including new and replacement phloem at 14 dpi with *C*Las-free psyllids. However, the *AtNPR1-OE* “Duncan” plants had larger xylem size than the WT (Fig. 3B). Upon inoculation with *C*Las-infected psyllids, we observed a substantial expansion of both phloem and xylem tissues in the WT “Duncan” plants, but only a moderate and significantly smaller expansion in the *AtNPR1-OE* “Duncan” plants (Fig. 3, A and B).

The sieve elements of plant stem sections were also observed with TEM. At 14 dpi with *C*Las-free psyllids, we detected a higher amount of callose deposition with visible sieve pore openings in the *AtNPR1-OE* “Duncan” stem sections compared with the WT sections that had no significant callose deposition (Fig. 3, C and D). Upon inoculation with *C*Las-infected psyllids, we observed tremendous callose accumulation in the WT “Duncan” stem sections with almost no visible pore openings, whereas the *AtNPR1-OE* “Duncan” plants had a much smaller reduction in pore openings caused by callose accumulation (Fig. 3, C and D).

### *AtNPR1* suppresses *C*Las-induced SA accumulation in citrus

**Figure 4.**
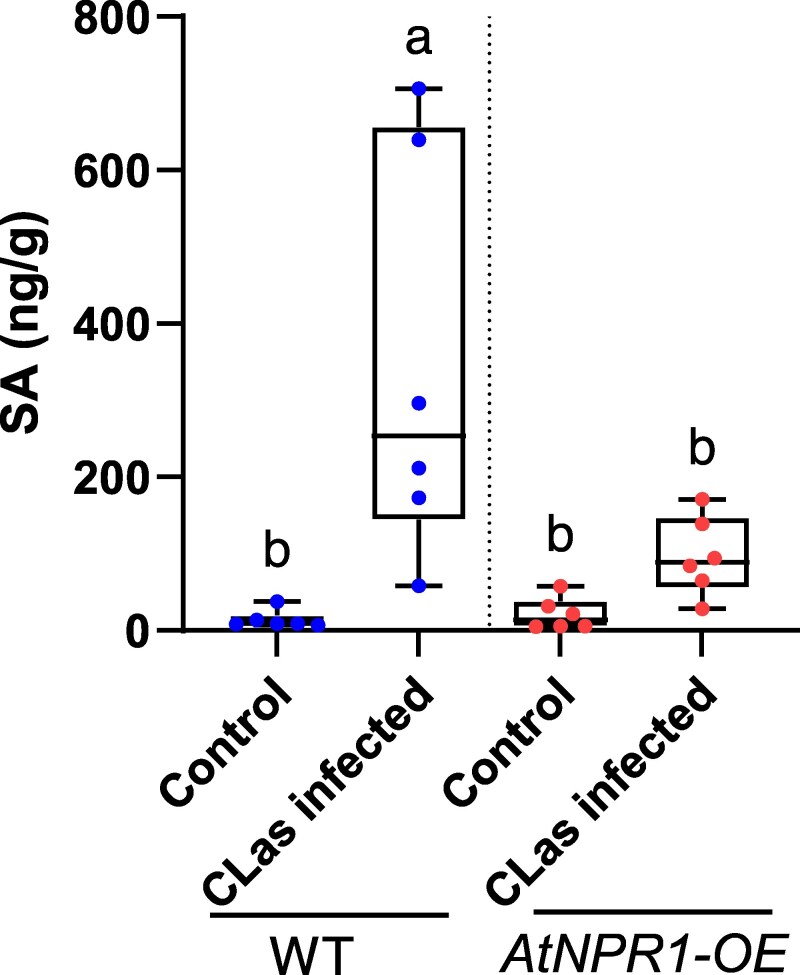
Citrus plants overexpressing *AtNPR1* reduce *C*Las-induced salicylic acid accumulation. Salicylic acid (SA) levels in WT and *AtNPR1*-overexpressing (*AtNPR1-OE*) “Duncan” citrus plants with and without *C*Las infection citrus plants maintained under greenhouse conditions for 1 yr. Data are presented as boxplots (The box extends from the first to the third quartile), showing median values (horizontal line at the center of the box) and individual data points (dots) with *n* = 6. The whiskers represent minimum and maximum values with no outliers. Different letters indicate statistically significant differences (*P* < 0.05) based on one-way ANOVA with Tukey test.

To investigate the role of *AtNPR1* in the regulation of SA levels, we quantified free SA accumulation in both *AtNPR1-OE* and WT “Duncan” citrus plants infected by *C*Las for a year. Healthy and *C*Las-infected plants were analyzed to compare SA levels in both genotypes. As shown in Fig. 4, WT “Duncan” plants exhibited a significant increase in SA levels with *C*Las infection. In contrast, while *AtNPR1-OE* “Duncan” plants also showed a mild increase in SA upon infection, the magnitude of SA accumulation was significantly lower than in the WT. These results suggest that *AtNPR1* suppresses *C*Las-induced SA accumulation in citrus, potentially contributing to its modulation of the plant's defense response.

### Silencing of *CsNPR3* suppresses *C*Las-induced callose and ROS accumulation and confers HLB tolerance

**Figure 5.**
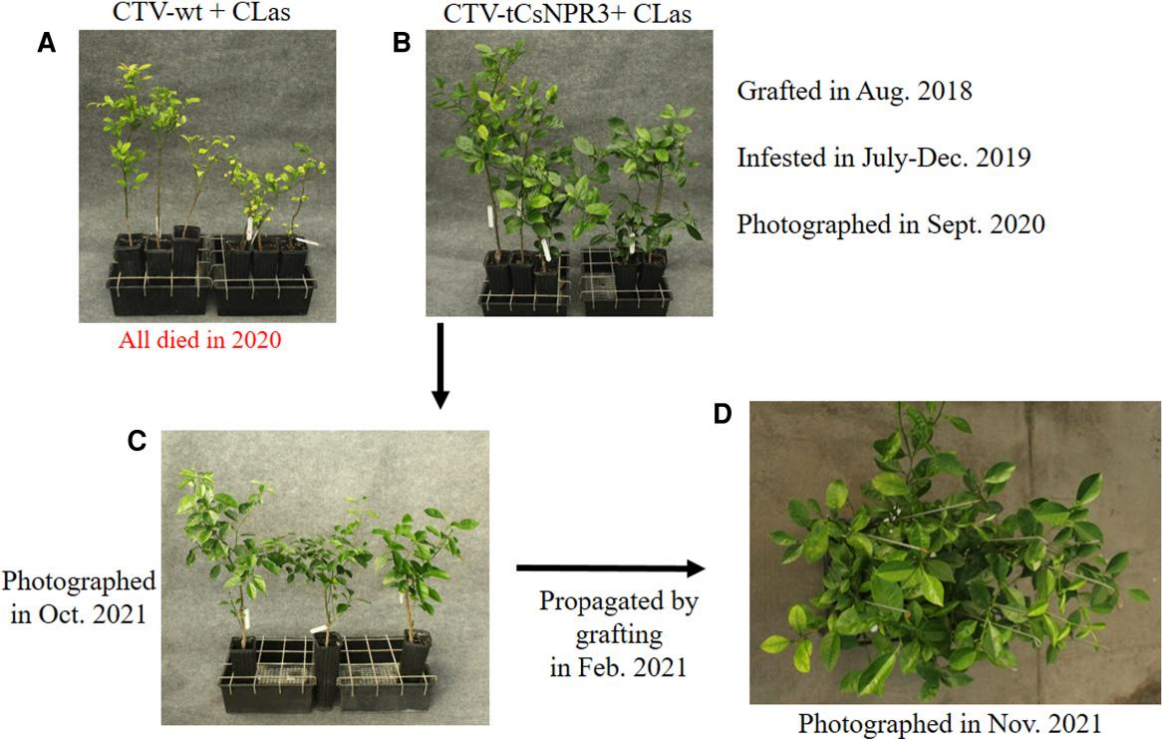
HLB symptom development in *C*Las-infected citrus *CsNPR3* RNAi plants. The wild-type CTV (CTV-wt) and CTV-tCsNPR3 (truncated *CsNPR3*) constructs were graft-inoculated into susceptible “Madam Vinous” sweet orange in August 2018. The “Madam Vinous” plants carrying the CTV constructs were infested with *C*Las-infected psyllids from July to December 2019. Photos were taken in September 2020. All CTV-wt plants infected by *C*Las died in 2020. The *C*Las-positive CTV-tCsNPR3 plants (*CsNPR3* RNAi lines) were propagated in February 2021. Photos of the parents and progenies were taken in November 2021. **A)** HLB symptoms on *C*Las-positive “Madam Vinous” plants carrying CTV-wt. **B)** HLB symptoms on *C*Las-positive “Madam Vinous” plants carrying CTV-tCsNPR3. **C)** The parental *C*Las-positive CTV-tCsNPR3 plants that provided budwoods for propagation. **D)** HLB symptoms on the *C*Las-positive CTV-tCsNPR3 progeny plants.

**Figure 6.**
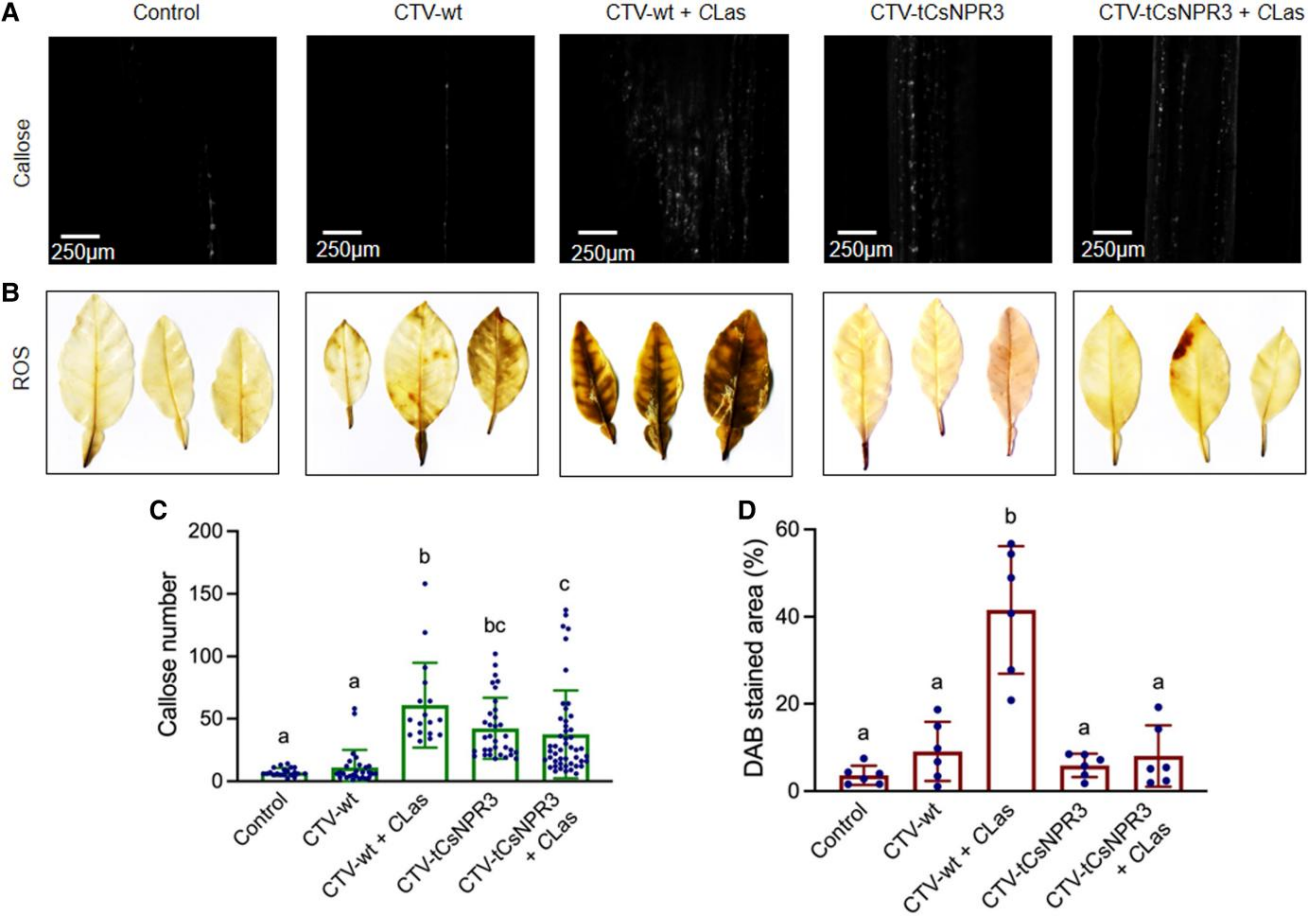
*C*Las-induced callose and ROS accumulation in citrus *CsNPR3* RNAi plants. **A)** Callose deposition revealed by aniline blue staining in CTV-wt, *C*Las-positive CTV-wt, CTV-tCsNPR3, and *C*Las-positive CTV-tCsNPR3 “Madam Vinous” stems. Representative images are shown. Control: healthy “Madam Vinous” stems. **B)** ROS accumulation revealed by DAB staining in CTV-wt, *C*Las-positive CTV-wt, CTV-tCsNPR3, and *C*Las-positive CTV-tCsNPR3 “Madam Vinous” leaves. Control: healthy “Madam Vinous” leaves. (Scale = 1 cm, images in the box are photographed at the same time). **C)** Numbers of callose depositions in the control and the CTV-wt, *C*Las-positive CTV-wt, CTV-tCsNPR3, and *C*Las-positive CTV-tCsNPR3 “Madam Vinous” stems. Bars represent “means ± Sd” (*n* = 17 to 48). Data from 3 independent experiments were combined. Different letters above the bars denote significant differences (*P* < 0.05; one-way ANOVA with Tukey's test). **D)** Percentages of leaf areas stained with DAB in the control and the CTV-wt, *C*Las-positive CTV-wt, CTV-tCsNPR3, and *C*Las-positive CTV-tCsNPR3 “Madam Vinous” leaves. Bars represent “means ± Sd” (*n* = 6). Different letters above the bars denote significant differences (*P* < 0.05; one-way ANOVA with Tukey's test).

Since CsNPR3 is the closest citrus ortholog of the AtNPR3 and AtNPR4 proteins (Supplementary Fig. S3), we tested whether the *CsNPR3* gene plays a role in *C*Las-induced callose and ROS accumulation and HLB symptom development. To this end, we used CTV-RNAi to silence the *CsNPR3* gene in citrus. Inoculation of a CTV-tCsNPR3 (truncated *CsNPR3*) construct into *Citrus macrophylla* efficiently silenced the *CsNPR3* gene (Supplementary Fig. S4). The CTV-tCsNPR3 construct was subsequently graft-inoculated into susceptible “Madam Vinous” sweet orange. The *CsNPR3* gene was also efficiently silenced in the CTV-tCsNPR3 “Madam Vinous” plants (Supplementary Fig. S4). “Madam Vinous” plants carrying the wild-type CTV (CTV-wt) and the CTV-tCsNPR3 construct were inoculated with *C*Las-infected psyllids. After becoming *C*Las positive, these plants were kept in a greenhouse for symptom development. Nine months later, the “Madam Vinous” plants carrying CTV-wt all exhibited severe HLB symptoms, whereas those with CTV-tCsNPR3 displayed no or mild symptoms (Fig. 5, A and B). We then determined callose and ROS levels in these plants and the control plants (with or without CTV and no *C*Las). Interestingly, silencing of *CsNPR3* elevated basal callose levels and suppressed *C*Las-induced callose deposition and ROS accumulation (Fig. 6, A to D). In these *C*Las-infected plants, expression levels of *CsCalS3* and *CsCalS7* were significantly lower in the CTV-tCsNPR3 plants compared with the CTV-wt plants (Supplementary Fig. S5). *CsRBOHD* expression levels were also downregulated, though not significantly (Supplementary Fig. S5). We next propagated the *CsNPR3* RNAi lines by grafting to test whether the HLB tolerance could be retained in the progeny. Nine months after the propagation, the progeny plants showed varying degrees of symptoms, ranging from no symptoms to visible symptoms (Fig. 5, C and D). This is likely due to varying silencing efficiency between flushes (Hajeri et al. 2014), resulting in segregation among the progeny plants screened for the HLB phenotype. These results together indicate that silencing of *CsNPR3* created HLB tolerance in susceptible “Madam Vinous” by repressing *C*Las-induced overaccumulation of callose and ROS. Thus, *CsNPR3* plays a positive role in HLB disease symptom development.

### *Arabidopsis* plants overexpressing *AtNPR1-OE* or carrying *npr3 npr4* double mutations exhibit increased basal callose levels but show suppressed callose deposition and ROS accumulation in response to *Psm* infection

**Figure 7.**
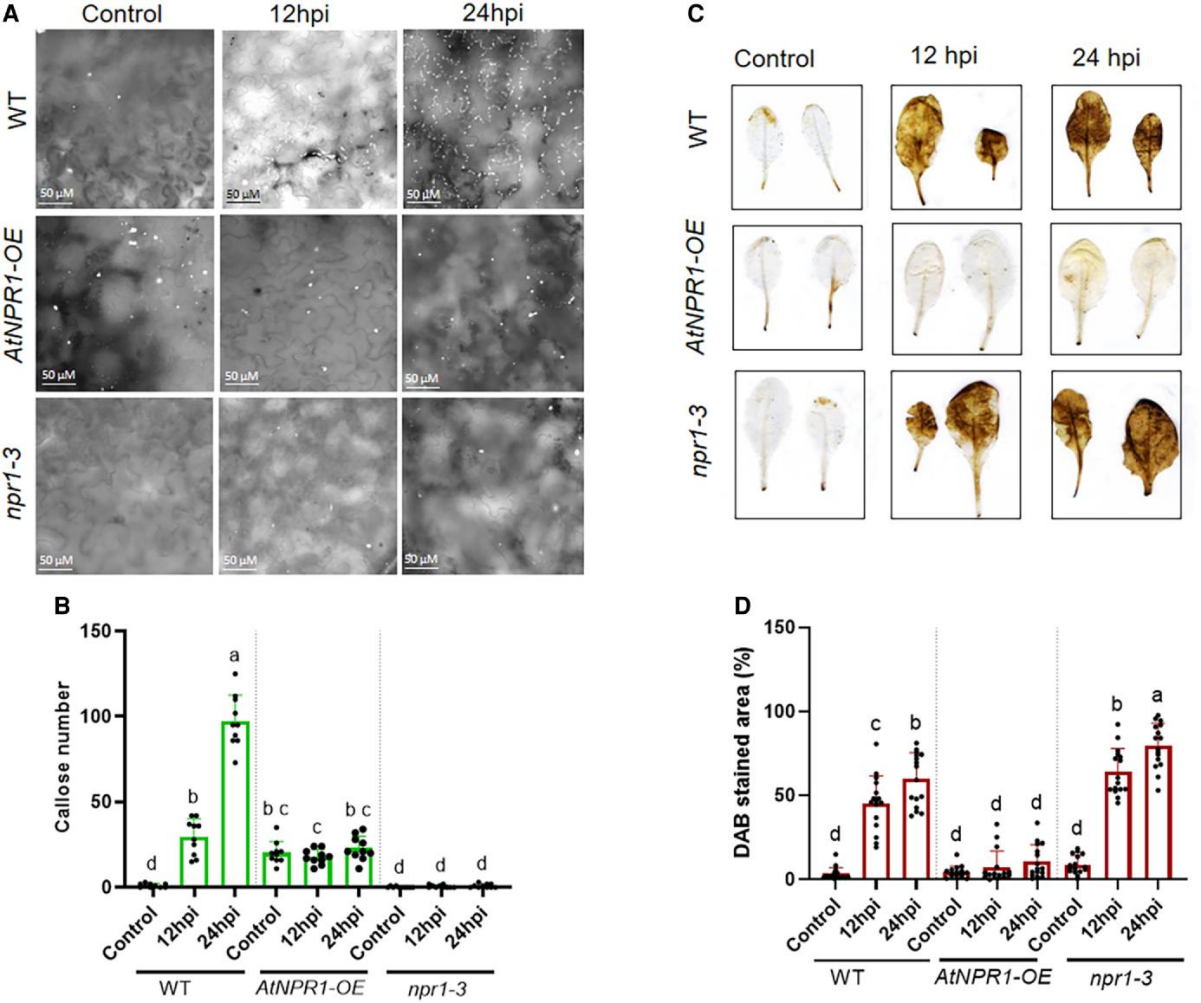
*Psm*-induced callose and ROS accumulation in *Arabidopsis AtNPR1-OE* and *npr1-3* plants. **A)** Callose deposition (white dots) revealed by aniline blue staining in *Arabidopsis* WT, *AtNPR1-OE*, and *npr1-3* leaves infected with *Psm* at 12 and 24 hpi. Representative images are shown. Control: healthy, uninfected *Arabidopsis* leaves. **B)** Number of callose depositions in the control and the *Arabidopsis* WT, *AtNPR1-OE*, and *npr1-3* leaves infected with *Psm* at 12 and 24 hpi. Bars represent “means ± Sd” (*n* = 10). Data from 3 independent experiments were combined. Different letters above the bars denote significant differences (*P* < 0.05; one-way ANOVA with Tukey's test). **C)** ROS accumulation revealed by DAB staining in *Arabidopsis* WT, *AtNPR1-OE*, and *npr1-3* leaves infected with *Psm* at 12 and 24 hpi. Representative images are shown (Scale = 0.5 cm, images in box photographed at the same time). Control: uninoculated healthy *Arabidopsis* leaves. **D)** Percentages of leaf areas stained with DAB in the control and the *Arabidopsis* WT, *AtNPR1-OE*, and *npr1-3* leaves infected with *Psm* at 12 and 24 hpi. Bars represent “means ± Sd” (*n* = 16 to 17). Data from 3 independent experiments were combined. Different letters above the bars denote significant differences (*P* < 0.05; one-way ANOVA with Tukey's test).

To understand if *AtNPR1*-mediated pathogen tolerance is conserved in other plant species, we opted to investigate the effects of *AtNPR1* in *Arabidopsis*. We compared the bacterial pathogen *Psm*-induced callose and ROS accumulation in WT *Arabidopsis*, *AtNPR1-OE Arabidopsis*, and *npr1-3* mutant plants. Interestingly, the *AtNPR1-OE Arabidopsis* plants accumulated significantly higher basal levels of callose than the WT and *npr1-3*, and *Psm* infection dramatically elevated callose deposition in the WT but did not significantly change callose levels in the *AtNPR1-OE Arabidopsis* and *npr1-3* plants (Fig. 7, A and B). These results revealed a paradox in which the physiologic low level of AtNPR1 is required for *Psm*-induced callose deposition, whereas ectopic expression of high levels of AtNPR1 increases basal callose levels but suppresses *Psm*-induced callose deposition. Furthermore, *Psm* infection triggered massive ROS accumulation, as measured by 3,3′-diaminobenzidine (DAB) staining, in both the WT and *npr1-3*, and the ROS levels at 12 and 24 h post-inoculation (hpi) are significantly higher in *npr1-3* than those in the WT, indicating that AtNPR1 negatively regulates *Psm*-induced ROS accumulation (Fig. 7, C and D). In line with this conclusion, *Psm*-induced ROS accumulation was almost completely suppressed in the *AtNPR1-OE Arabidopsis* plants (Fig. 7, C and D). These results indicate that AtNPR1 is a major regulator of *Psm*-induced callose deposition and ROS accumulation in *Arabidopsis*. We also analyzed *Psm*-induced callose deposition and ROS accumulation in 2 previously described *Arabidopsis npr3 npr4* double mutants (Zhang et al. 2006). Although *Psm* significantly induced callose deposition in the *npr3-1 npr4-3* mutant, the induction level was significantly lower than that in the WT plants (Supplementary Fig. S6), indicating that *AtNPR3* and *AtNPR4* positively regulate *Psm*-induced callose deposition. Furthermore, both *npr3-2 npr4-2* and *npr3-1 npr4-3* accumulated elevated basal ROS levels, and *Psm* infection did not further increase ROS levels in the *npr3-1 npr4-3* mutant (Supplementary Fig. S6), suggesting that *AtNPR3* and *AtNPR4* suppress basal ROS and may positively contribute to pathogen-induced ROS accumulation.

## Discussion

In this study, we investigated the role of *NPR* genes in regulating basal and pathogen-induced callose and ROS accumulation in citrus and *Arabidopsis*. Our results show that overexpression of *AtNPR1* in both citrus and *Arabidopsis*, and silencing *CsNPR3* in citrus or knocking out *AtNPR3/4* in *Arabidopsis*, similarly elevate basal callose levels and suppress pathogen-induced callose and ROS accumulation. These results in HLB tolerance in citrus, suggesting that overexpression of *AtNPR1* and silencing of *CsNPR3* are promising approaches to mitigating the HLB disease.

*C*Las infection in most commercial citrus varieties causes chronic immune imbalance, leading to excessive accumulation of callose and ROS in the phloem. This imbalance triggers phloem plugging, hindering the export of photoassimilates, resulting in starch accumulation and the manifestation of HLB symptoms (Koh et al. 2012; Deng et al. 2019; Achor et al. 2020; Ma et al. 2022; Welker et al. 2022; Yao et al. 2023). Interestingly, some citrus varieties and relatives exhibit little or no HLB symptoms after being infected by *C*Las (Wang 2021; Estrella-Maldonado et al. 2023). Tolerant citrus varieties display increased induction of genes encoding key immune regulators and enzymes that can degrade callose and ROS, preventing their overaccumulation (Weber et al. 2022). These findings suggest that HLB-susceptible varieties lack the necessary capacity to prevent *C*Las-induced callose and ROS overaccumulation.

Callose levels are generally regulated by a balance of synthesis and removal (German et al. 2023). During pathogen infection, both callose biosynthetic genes and catabolic genes like *PR2*, which encodes a β-1,3-glucanase that degrades callose, are induced (Oide et al. 2013). The overaccumulation of callose in the phloem due to *C*Las infection may result from heightened induction of callose biosynthetic genes and/or compromised induction of catabolic genes (Fig. 2E). NPR1 appears to play a crucial role in pathogen-induced callose deposition (Dong et al. 2008; Wang et al. 2013, 2021; Chen et al. 2017). While overexpression of *AtNPR1* results in elevated basal callose levels, pathogen-induced callose deposition is significantly reduced in *AtNPR1-OE* citrus and *Arabidopsis* plants (Figs. 2, A and B, 3, C and D, and 7, A and B). This reduction in pathogen-induced callose deposition likely enhances plant survival by mitigating the adverse effects of callose overaccumulation.

ROS accumulation is also tightly regulated through a balance between production and scavenging. Pathogen-induced ROS production is primarily mediated by plasma membrane-localized RBOHs (Torres et al. 2002), whose activity is precisely controlled by phosphorylation and S-nitrosylation, which are concomitantly activated in response to pathogen infection (Yun et al. 2011; Kadota et al. 2014; Kimura et al. 2020; Lee et al. 2020). ROS can be scavenged by antioxidant enzymes such as catalase, glutathione/ascorbate peroxidase, and glutathione S-transferase (You and Chan 2015). Upon pathogen infection, the activities of these enzymes are significantly upregulated, ensuring that ROS levels subside after the initial oxidative burst (Sahu et al. 2022). Therefore, *C*Las-triggered overaccumulation of ROS in susceptible citrus varieties is likely due to impaired negative regulation of ROS production and/or compromised induction of antioxidant enzymes.

NPR1 plays a crucial role in regulating ROS production and scavenging. Overexpression of *NPR1* genes in various plant species reduces ROS levels and enhances ROS scavenging activity during pathogen infection (Xu et al. 2019; He et al. 2023). Overexpression of *AtNPR1* drastically inhibits *C*Las- and *Psm*-induced ROS accumulation in citrus and *Arabidopsis*, respectively (Figs. 2, C and D and 7, C and D), while *Arabidopsis npr1* mutant plants accumulate higher levels of ROS in response to *Psm* (Fig. 7, C and D). These findings demonstrate that *NPR1* prevents pathogen-induced ROS overaccumulation and alleviates its toxic effects.

Based on these findings, we carried out vascular anatomy using microscopy. There was no difference in phloem size between *AtNPR1*-overexpressing and non-transgenic citrus trees. However, it is worth noting that the xylem fiber length was observed to be larger in the transgenic trees (Fig. 3). Increased xylem vessels have been observed previously with tolerant citrus varieties, where larger xylem vessels are linked to increased leaf transpiration rate, leaf biomass, and increased root hydraulics, thereby balancing water deficit caused by HLB (Rodríguez-Gamir et al. 2010; Hamido et al. 2019; Robledo et al. 2024). In response to drought stress, plants synthesize abscisic acid (ABA) in the roots, where water deficit is sensed, and transport ABA via the xylem to the shoots to regulate stomatal closure and other adaptive responses. Therefore, maintaining xylem integrity and function may not only support water transport but also facilitate systemic drought signaling under HLB-induced stress (Li et al. 2023). Upon *C*Las-exposure (*C*Las + 14 dpi), we observed a subtle increase in the phloem size and xylem size in the transgenic *AtNPR1-OE* trees, unlike the robust change in the vascular bundle dynamics in the non-transgenic trees (Fig. 3). This can again be associated with the ability of *AtNPR1-OE* plants to maintain their basal levels of immune responses.

We found that silencing *CsNPR3* can offer a viable alternative for developing HLB-tolerant citrus varieties. Silencing *CsNPR3* in susceptible “Madam Vinous” sweet orange increases basal callose levels, inhibits *C*Las-induced callose and ROS accumulation, and enhances HLB tolerance (Figs. 5 and 6). Similarly, in *Arabidopsis*, *npr3 npr4* mutants exhibit elevated basal ROS levels and inhibited *Psm*-induced callose deposition and ROS accumulation (Supplementary Fig. S6), indicating that *NPR3* and *NPR4* contribute to pathogen-induced callose and ROS accumulation. Thus, *CsNPR3* plays a positive role in HLB symptom development, and silencing it mimics the effects of *AtNPR1* overexpression, making it a promising target for mitigating HLB symptoms.

**Figure 8.**
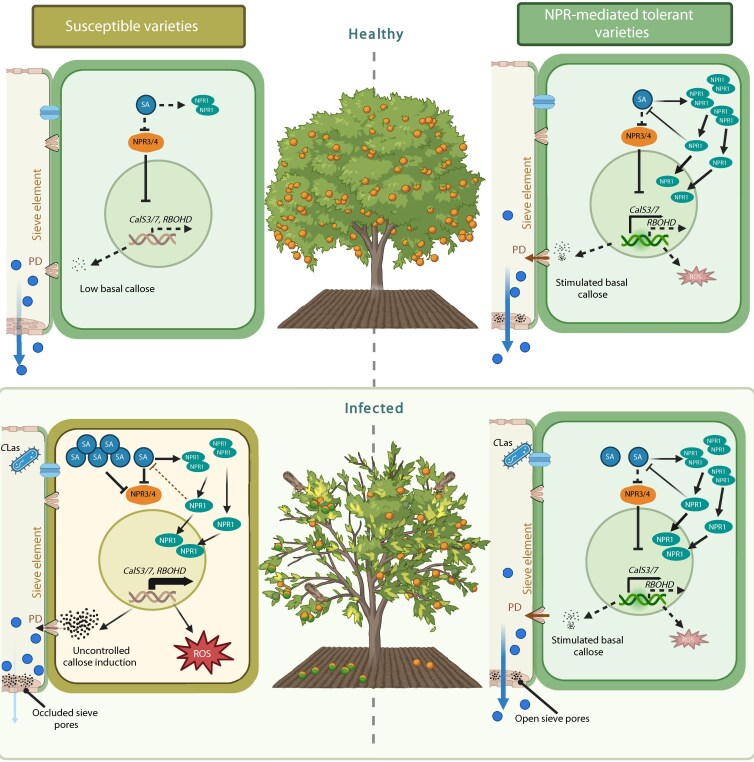
Proposed mechanism of NPR-mediated tolerance in citrus. In healthy susceptible varieties (upper left), low basal levels of SA result in minimal transcription of defense-related genes, including callose synthases (*CalS3*, *CalS7*) and ROS-related genes, such as *respiratory burst oxidase homolog D* (*RBOHD*). In infected susceptible plants (lower left), excessive SA induction leads to simultaneous activation of NPR1 and suppression of NPR3/4, causing increased transcription of defense-related genes (*CalS3/7*, *RBOHD*, etc.). This results in uncontrolled accumulation of callose and oxidative stress (ROS), leading to sieve element occlusion and reduced nutrient flow (light blue arrow). Healthy *AtNPR1*-*OE* plants (upper right) have elevated levels of NPR1, which increases basal levels of defense-related genes, such as *CalS3/7*, while modulating SA levels to minimize *RBOHD* transcription. In infected *AtNPR1*-*OE* plants (lower right), elevated levels of NPR1 suppress SA accumulation, resulting in no significant changes in the transcription levels of callose and ROS-related genes, which in turn prevent complete sieve plate occlusion. Consequently, immune balance is maintained, and nutrient flow is ensured (blue arrow). (Image created with BioRender.com).

We propose that HLB disease susceptibility is linked to immune imbalance in susceptible citrus varieties, which could be rectified by *NPR1* overexpression or *NPR3* repression (Fig. 8). NPR1 likely maintains immune homeostasis through feedback inhibition of SA biosynthesis, preventing excessive SA accumulation upon infection (Fig. 4), thereby suppressing the over-induction of callose and ROS biosynthesis-related genes (Lee et al. 2011; Zavaliev et al. 2020) (Fig. 8). This model is supported by the observation that HLB-infected susceptible citrus varieties demonstrate induced levels of SA (Nehela et al. 2018; Peng et al. 2021; Ibanez et al. 2022), whereas tolerant citrus varieties and *AtNPR1*-*OE* plants accumulate reduced SA levels, consistent with the role of NPR1 as a negative regulator of SA biosynthesis (Fig. 4) (Delaney et al. 1995; Friedrich et al. 2001; Fitzgerald et al. 2004; Suh et al. 2021). Therefore, balanced immune responses in *AtNPR1-OE* plants likely result from NPR1-mediated elevation of basal transcription of defense-related genes (*CalS3/7*, *RBOHD*, etc.) and inhibition of SA accumulation. In *CsNPR3* RNAi plants, NPR3-mediated transcriptional repression and NPR1 degradation are probably alleviated (Fu et al. 2012; Ding et al. 2018; Ramasamy et al. 2024), leading to elevated transcription of defense genes and accumulation of CsNPR1, mimicking the effects of *AtNPR1* overexpression in the plants.

Our findings underscore the crucial role of proper immune equilibrium in protecting crop plants against pathogens and show that the immune defects in HLB-susceptible varieties, which lead to uncontrolled callose and ROS accumulation, could be mitigated through overexpression of *AtNPR1* or silencing of *CsNPR3*. While our study focused on *AtNPR1* to leverage its well-characterized role in regulating SAR, future work should evaluate whether overexpression of the citrus homolog, *CsNPR1*, can provide similar benefits. Overall, our work suggests that HLB stems from an immune imbalance in the susceptible citrus varieties and that genetic approach to enhance plant immunity and maintain immune homeostasis can correct this defect to generate disease tolerance.

## Materials and methods

### Plant materials

Budwood of the previously reported *AtNPR1*-*OE “*Hamlin” sweet orange line 13-3 and “Duncan” grapefruit line 57-28 (Zhang et al. 2010) and the corresponding non-transgenic WTs were grafted onto “Volkamer” lemon rootstock using a wedge-grafting method. All progeny plants were grown in a greenhouse with controlled temperature and humidity of 25 °C and 60%, respectively. The grafted plants were used 2 mo later for *C*Las inoculation experiments. *Arabidopsis* plants used in this study are WT Col-0, *AtNPR1-OE* in Col-0 background (Cao et al. 1998), *npr1-3* (Cao et al. 1997), *npr3-2 npr4-2*, and *npr3-1 npr4-3* (Zhang et al. 2006).

To create *CsNPR3* RNAi plants, a cDNA fragment of *CsNPR3* was amplified with the primers *StuI-tCsNPR3F* and *PacI-tCsNPR3R* (Supplementary Table S1) and cloned into pCAMBIA1380 with p22 of ToCV using PacI and StuI at the 3′ end of CTV under the duplicated CP-CE of CTV as previously described (Hajeri et al. 2014). The empty vector was used as a control (CTV-wt). The constructs were introduced into *Nicotiana benthamiana* and subsequently bark-snap inoculated into *C. macrophylla* as described (Hajeri et al. 2014). After systemic infection in the *C. macrophylla* plants was confirmed by enzyme-linked immunosorbent assay (ELISA) and silencing of *CsNPR3* was validated by RT-qPCR, the constructs were graft-transmitted into “Madam Vinous” sweet orange. After systemic infection in the “Madam Vinous” plants was verified by ELISA, the plants were inoculated with *C*Las-infected psyllids in a containment growth room as described previously (Robertson et al. 2018). Once the plants were tested *C*Las positive, they were maintained in a clean growth room for HLB symptom development. Three plants from each group were used as biological replicates for each experiment.

### Insects

Populations of healthy and *C*Las-infected Asian citrus psyllids (*Diaphorina citri*) were maintained in cages in controlled rooms on curry leaf plants (*Bergera koenigii*) and *C. macrophylla* plants, respectively. The populations were tested periodically for the presence of *C*Las by qPCR with *C*Las-specific primers (Supplementary Table S1).

### *C*Las and *Psm* infection

For *C*Las infection of citrus, a single leaf, still attached to each plant, was placed in a closed chamber (a clear plastic cup with a dome lid) and 10 *C*Las-free or 10 *C*Las-infected psyllids were released into the chamber to feed on the leaf. The leaves and stem-phloem bark adjacent to the inoculated leaves were harvested at the indicated time points for callose analysis, DAB staining, SA analysis, as well as gene expression. At least 6 plants were used for this experiment. For *Psm* infection of *Arabidopsis*, an overnight culture of the bacterial pathogen *Psm* with an OD_600_ of 0.2 was used. The culture was diluted in 1 mm MgCl_2_ to an OD_600_ of 0.001, and the diluted *Psm* suspension was infiltrated into fully expanded leaves on 4-wk-old *Arabidopsis* plants. The 1 mm MgCl_2_ solution was used as a mock inoculation control. The infiltrated leaves were collected at 12 and 24 hpi for callose and ROS detection.

### Callose detection

For callose detection in the phloem, the stem bark adjacent to the psyllid-infested leaves was peeled (1 to 2 cm long) using a sharp scalpel, ensuring that the phloem portion was included (Supplementary Fig. S1). At least 3 citrus trees were used as biological replicates. The samples were snap-chilled in 85% ice-cold ethanol, followed by fixation overnight in the same solution at room temperature. For *Arabidopsis*, the *Psm*-infiltrated leaves were detached from the plants at 12 and 24 hpi and dipped in chilled 85% ethanol, followed by overnight fixation. The ethanol was removed the next day, and the samples were washed with 0.01% Tween 20 for 1 h, followed by staining with 0.1% aniline blue (in 10 mm potassium phosphate buffer, pH 12) in the dark for at least 1 h. The samples were visualized under a Leica SP8 confocal microscope with an excitation wavelength of 405 nm and a band-pass 420 to 480 nm emission filter (Supplementary Fig. S1). The number of callose deposits per 10× (40× for *Arabidopsis*) field was detected using a modified macrocode in ImageJ software (Zavaliev and Epel 2015; Welker and Levy 2022).

### Detection of ROS

The infected leaves were collected and immediately submerged in a freshly made DAB solution (1 mg/mL, pH 3.0) and incubated overnight on a shaker at 50 rpm. The leaf samples were washed twice in a destaining solution (ethanol/glycerol/acetic acid, ratio 3/1/1), each for 20 min at 95 °C and room temperature, respectively. The leaf samples were scanned, and the staining area was measured in ImageJ using the Color Devonvolution2 plugin and the H DAB vector. The percentage of leaf area with brown stain was calculated using the equation: % DAB-stained area = (stained leaf area/total leaf area) × 100.

### SA detection using liquid chromatography-mass spectrometry (LC-MS)

Leaf samples (80 to 100 mg) were collected and snap-frozen in liquid N_2_ and homogenized using the TissueLyserII (Qiagen). SA was extracted from the tissue samples using cold methanol:acetonitrile (50:50, v/v) spiked with deuterium-labeled internal standards (mixture of D4-SA). After centrifugation at 16,000 *g*, the supernatants were collected, and the extraction of pellet was repeated 1 more time. The pellets were dried and re-dissolved in 15% methanol and run using an LC-MS MRM (Multiple Reaction Monitoring) targeted assay, as previously detailed in Lopez-Guerrero et al. (2022). Briefly, LC separation is done on a ZORBAX Eclipse Plus C18 column (2.1 mm × 100 mm, Agilent), flowing at 0.45 mL/min. The gradient of the mobile phases A (0.1% formic acid) and B (0.1% formic acid/90% acetonitrile) was as follows: 5% B for 1 min, to 60% B in 4 min, to 100% B in 2 min, hold at 100% B for 3 min, to 5% B in 0.5 min. The Shimadzu LC system was interfaced with a Sciex QTRAP 6500+ mass spectrometer equipped with a TurboIonSpray (TIS) electrospray ion source. The instrument was set up to acquire data in negative and positive ion modes. Analyst software (version 1.6.3) was used to control sample acquisition and data analysis. SA was detected using MRM transitions that were optimized using the standard. For quantification, an external standard curve was prepared using a series of standard samples containing different concentrations of unlabeled hormones and fixed concentrations of the deuterium-labeled standards mixture.

### RNA extraction and quantitative PCR (RT-qPCR)

Leaf samples (100 mg) were detached and immediately snap-frozen in liquid N_2_ and ground in a tissue lyser for RNA isolation using TRI RNA Isolation Reagent (Sigma) according to the manufacturer's instructions. The isolated RNA was used for cDNA preparation using the High-Capacity cDNA Reverse Transcription Kit (Applied Biosystems) according to the manufacturer's instructions. RT-qPCR was performed using SYBR Green PCR Master Mix (Applied Biosystems) on a 7500 Fast Real-Time PCR system (Applied Biosystems) according to the user's manual. The 2^−ΔΔCt^ method was used to determine the relative level of gene expression (Livak and Schmittgen 2001). The *CsGAPDH* gene was used as an internal control. The efficiency of the primers was determined as previously described (Pfaffl 2001). The primers are listed in Supplementary Table S1.

### Light microscopy and TEM

For both light microscopy and electron microscopy, the stems of each sample were cut into 1 cm pieces and immediately fixed in 3% (v/v) glutaraldehyde in 0.1 m potassium phosphate buffer (pH 7.2) for 4 h at room temperature. After washing with more phosphate buffer, the samples were fixed again in 2% osmium tetroxide for 4 h at room temperature. The samples were again washed with the same phosphate buffer. The samples were next dehydrated in a series of acetone solutions, starting at 10% and ending at 100%, increasing by 10% each stage, soaking in each solution for 10 min. The samples were placed in a 50% (v/v) Spurr's resin and acetone solution, then a 70% (v/v) Spurr's resin and acetone solution, each for 8 h. Lastly, the samples were embedded in 100% Spurr's resin by baking in a 70 °C oven overnight. For light microscopy, the samples were semithin-sectioned at a depth of 0.99 *µ*m. The sections were placed on a glass slide and heat sealed on a slide warmer. The cells in the section were stained with methylene blue, followed by counter stain in basic fuchsin to stain lignins and other hydrophobic compounds in the cells for 30 s each. The dried and mounted sections were imaged with a light microscope with an attached camera. The images were analyzed in ImageJ for measurement of phloem and xylem sizes. For electron microscopy, ultrathin sections of 90 nm were made and mounted onto 200-mesh formvar-coated copper grids, stained with Uranyless for 10 min, rinsed with distilled water, and stained with Reynold's lead citrate for 5 min. The grids were washed with distilled water and 0.1 N NaOH, then allowed to dry overnight. The samples were then observed with a Hitachi H-7650 transmission electron microscope with an attached AMT XR50 camera. Images obtained from the observations were analyzed in ImageJ, and the sieve plate openings were measured.

### Statistical analysis

Each experiment was repeated at least 3 times. The sample sizes (*n*) are mentioned in the figure legends. Statistical analyses were performed using the data analysis tools (Student's *t*-test) in Microsoft Excel of Microsoft Office 2023 for Macintosh, as well as one-way ANOVA in Prism 10.

### Accession numbers

Sequence data from this article can be found in the GenBank/Citrus genome data libraries under accession numbers as follows, AtNPR1: AT1G64280; AtNPR2: AT2G42395; AtNPR3: AT5G45110; AtNPR4: AT4G19660; CsNPR3: Cs2g10790, CsNPR1: Cs4g14600; CsCalS3: Cs5g14730; CsCalS7: Cs7g17340; CsRBOHD: Cs7g19320; CsGAPDH: Cs5g06870.2; CsActin: Cs7g16360; *C*Las-16sDNA: WP_012778365.1.

## Supplementary Material

kiaf532_Supplementary_Data

## Data Availability

The authors declare that all data supporting the findings of this study are available within the manuscript and its supplementary files. Source files are available from the corresponding authors upon request.

## Supplementary data

The following materials are available in the online version of this article.

**Supplementary Figure S1.** Structure of bark peels.

**Supplementary Figure S2.** Time-course analysis of *C*Las-induced ROS accumulation in citrus leaves.

**Supplementary Figure S3.** A phylogenetic tree of citrus NPR homologs, *At*NPR1, *At*NPR2, *At*NPR3, and *At*NPR4.

**Supplementary Figure S4.** Silencing of *Cs*NPR3 by CTV-RNAi.

**Supplementary Figure S5.** CsCalS3, CsCalS7, and CsRBOHD levels in *C*Las-infected CTV-tCsNPR3.

**Supplementary Figure S6.** Psm-induced callose and ROS accumulation in Arabidopsis npr3 npr4 plants.

**Supplementary Table S1.** Primers used in this study.

## Dive Curated Terms

The following phenotypic, genotypic, and functional terms are of significance to the work described in this paper:

NPR4 Gramene: AT4G19660
NPR4 Araport: AT4G19660

TRI Gramene: AT2G29330
TRI Araport: AT2G29330

rbohD Gramene: AT5G47910
rbohD Araport: AT5G47910

PR1 Gramene: AT2G14610
PR1 Araport: AT2G14610

leaf AmiGo: PO:0025034

Salicylic acid CHEBI: CHEBI:16914

CALS7 Gramene: AT1G06490
CALS7 Araport: AT1G06490
