# Supplementary material for: NPR1 suppresses Candidatus Liberibacter asiaticus-induced callose and reactive oxygen species accumulation
Source: Plant Physiol. 2025 Oct 21;199(3):kiaf532. doi: 10.1093/plphys/kiaf532 (PMC12631789; doi:10.1093/plphys/kiaf532)
Supplement: kiaf532_Supplementary_Data [file kiaf532_supplementary_data.zip › Supplementary Table resubmit.docx]

**Supplementary Table 1** Primers used in this study

| Primer name | Target | Sequence (5’ to 3’) |
| --- | --- | --- |
| CQULA04F | *C*Las- 16SrDNA | TGGAGGTGTAAAAGTTGCCAAA |
| CQULA04R |  | CCAACGAAAAGATCAGATATTCCTCTA |
| *C*Las probe |  | 6FAM-ATCGTCTCGTCAAGATTGCTATCCGTGATACTAG |
| M-1636 | *CsNPR3* | CGTATGGCAAGGTTGGATATGA |
| M-1637 |  | GTTGACACCTCCATCGGAAA |
| CsCalS7-F | *CsCalS7* | GACGCCTAACCGAGTACCTGC |
| CsCalS7-R |  | GTGCAGCTGGTGATCCATCA |
| CsCalS3-F | *CsCalS3* | GGCCTCCGTTCTTACTTGCT |
| CsCalS3-R |  | ACACTCCTTGACAGCACAGG |
| CsRBOHD-F | *CsRBOHD* | CCCTCGGCTTATAAATGCAA |
| CsRBOHD-R |  | CAAAAGGCATTGAACCCAGT |
| CsGAPDH-F | *CsGAPDH* | GGAAGGTCAAGATCGGAATCAA |
| CsGAPDH-R |  | CGTCCCTCTGCAAGATGACTCT |
| StuI-tCsNPR3F | *CsNPR3* fragment | TCTAGGCCTACGTCAGCATCTGTAGAAGATTGACAA |
| PacI-tCsNPR3R |  | ACCTTAATTAACAGGTGTCTCATTTAAGTCAACCTCCCTTAA |
| CsActin-F | *CsActin* | GTTGCCATTGGTTGGTATTTGATAC |
| CsAcrin-R |  | CGTCGACTGCCATTCCAGAT |
