# Supplementary material for: NPR1 suppresses Candidatus Liberibacter asiaticus-induced callose and reactive oxygen species accumulation
Source: Plant Physiol. 2025 Oct 21;199(3):kiaf532. doi: 10.1093/plphys/kiaf532 (PMC12631789; doi:10.1093/plphys/kiaf532)
Supplement: kiaf532_Supplementary_Data [file kiaf532_supplementary_data.zip › Supplementary figures re.pdf]

Supplementary Information

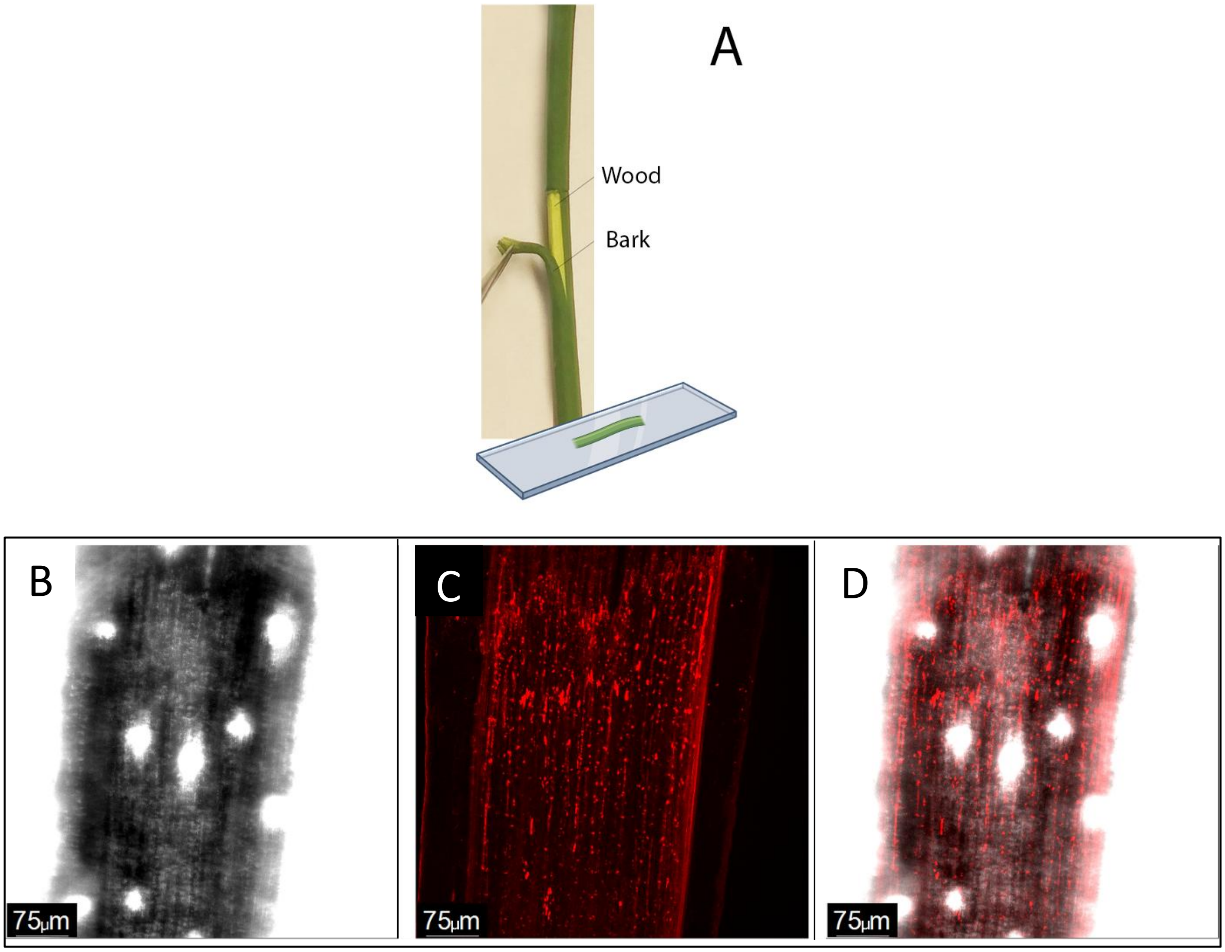

Supplementary Figure S1: ***Structure of Bark peels***

For callose staining, the bark tissue was peeled from the stem and mounted on the microscope slides (A). Pictures are takes from the inner side of the bark, where the phloem tissue can be visualized (B-D).

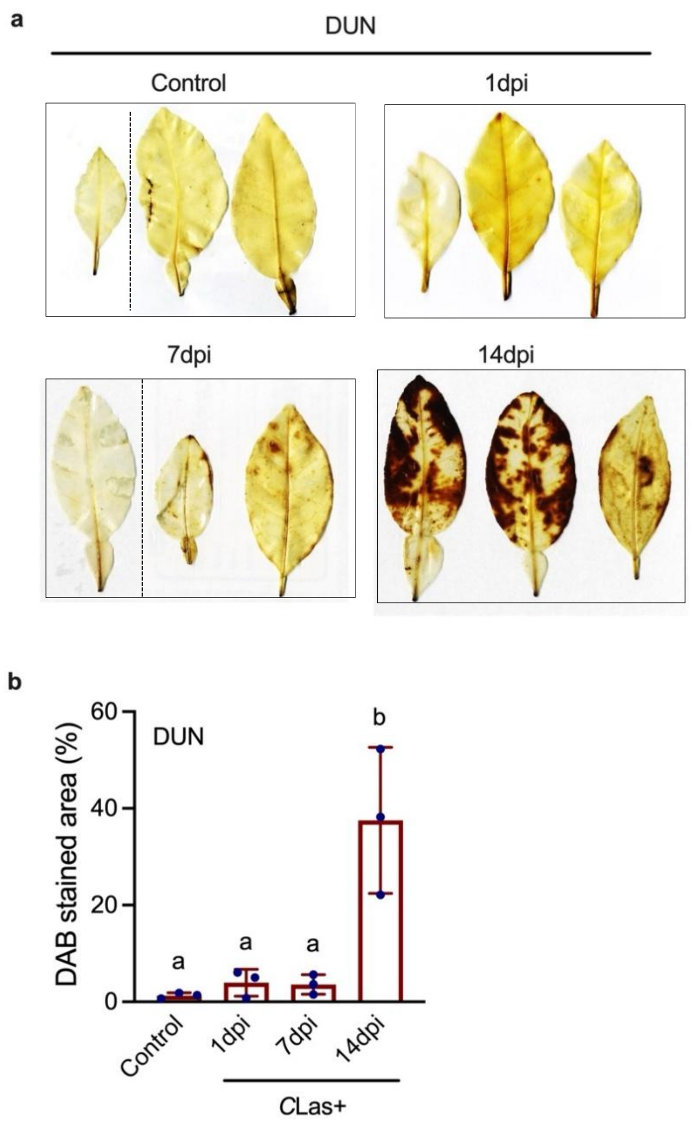

Supplementary Figure S2: ***Time-course analysis of CLas-induced ROS accumulation in citrus leaves***

a, ROS accumulation (brown precipitates) revealed by DAB staining in 'Duncan' grapefruit leaves inoculated with *C*Las-infected psyllids at 1 day post-inoculation (dpi), 7 dpi, and 14 dpi. Control: uninoculated healthy 'Duncan' leaves. (images in box photographed at the same time)

b, Percentages of leaf areas stained with DAB in the control and the 'Duncan' leaves inoculated with *C*Las-infected psyllids at 1, 7, and 14 dpi. Bars represent means ± SD (n = 3). Different letters denote significant differences ($p < 0.05$; one-way ANOVA with Tukey's test).

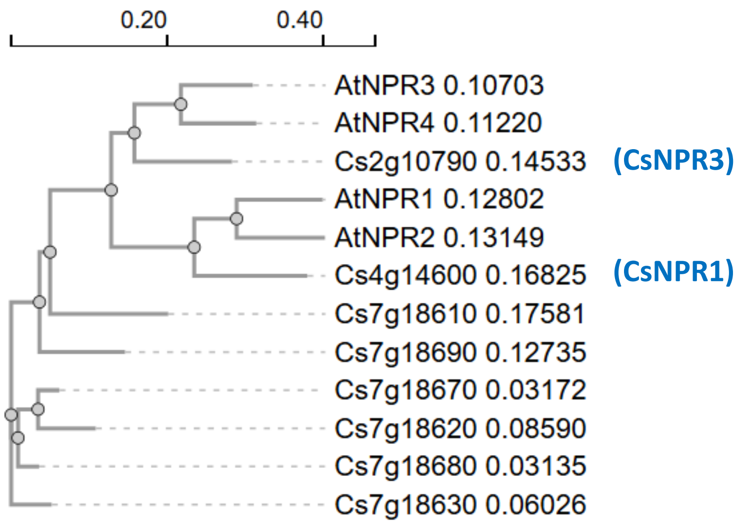

Supplementary Figure S3: ***A phylogenetic tree of citrus NPR homologs, AtNPR1, AtNPR2, AtNPR3, and AtNPR4***

The AtNPR1 amino acid sequence was used as the query sequence to BLAST the citrus genome database and the top nine hits plus the AtNPR proteins were used to generate the phylogenetic tree by Simple Phylogeny (https://www.ebi.ac.uk/jdispatcher/phylogeny/simple_phylogeny).

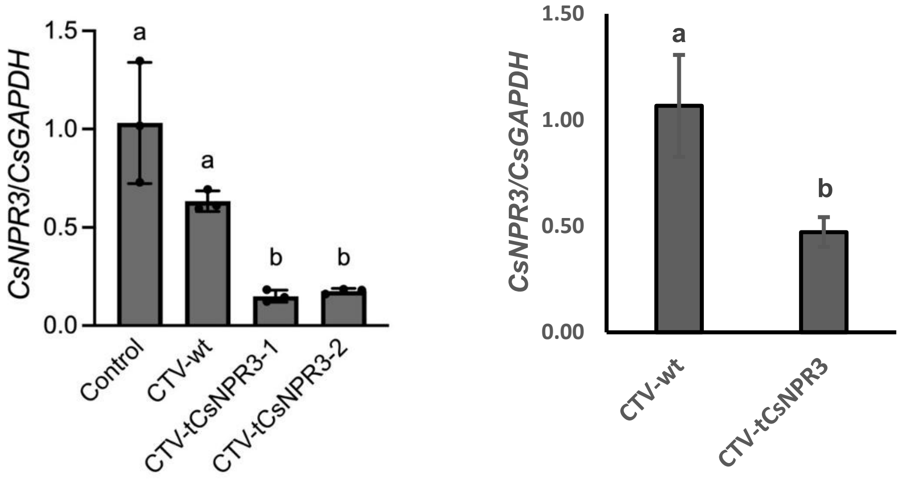

Supplementary Figure S4: ***Silencing of CsNPR3 by CTV-RNAi***

Left- Expression of *CsNPR3* in healthy (Control), CTV-wt, and CTV-tCsNPR3 *C. macrophylla* plants. Two CTV-tCsNPR3 lines were tested. Bars represent means ± SD (n = 3). Different letters denote significant differences ($p < 0.05$; one-way ANOVA with Tukey's test). *Right*- Expression of *CsNPR3* in CTV-wt and CTV-tCsNPR3 *C. sinensis* 'Madame Vinous' plants. Bars represent means ± SE (n = 3). Different letters denote significant differences ($p < 0.05$; t-test).

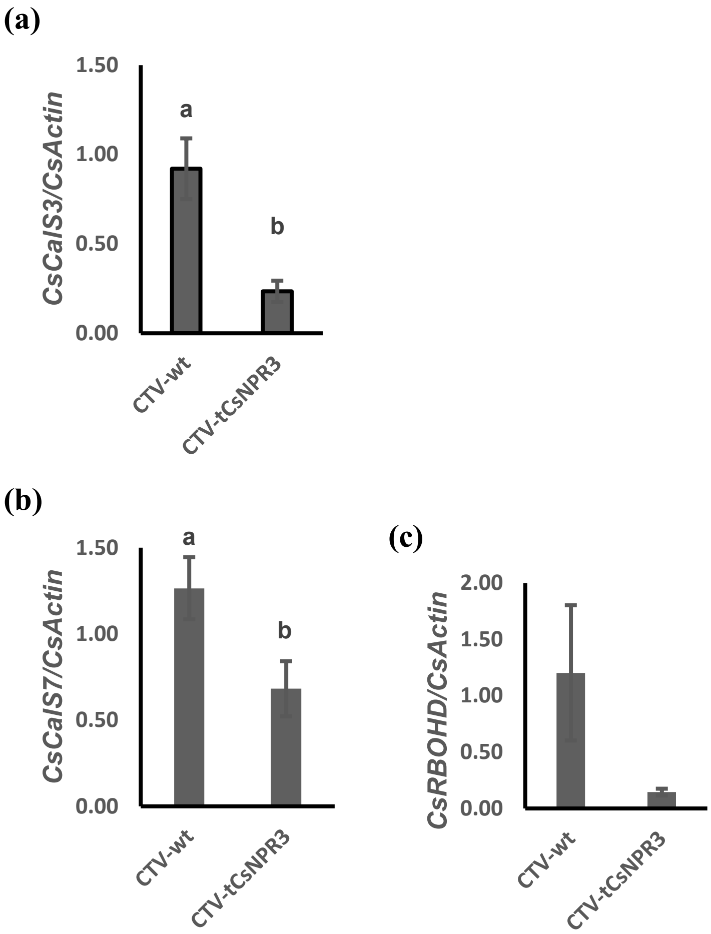

Supplementary Figure S5: *CsCalS3*, *CsCalS7*, and *CsRBOHD* levels in CLas infected CTV-tCsNPR3

Expression of *CsCalS3* (a), *CsCalS7* (b), and *CsRBOHD* (c) in CTV-WT and CTV-tCsNPR3 'Madame Vinous' sweet orange leaves inoculated with *C*Las-infected psyllids. Bars represent 'means ± standard error (SE)' (n = 3). Different letters above the bars denote significant differences ($p < 0.05$; t-test).

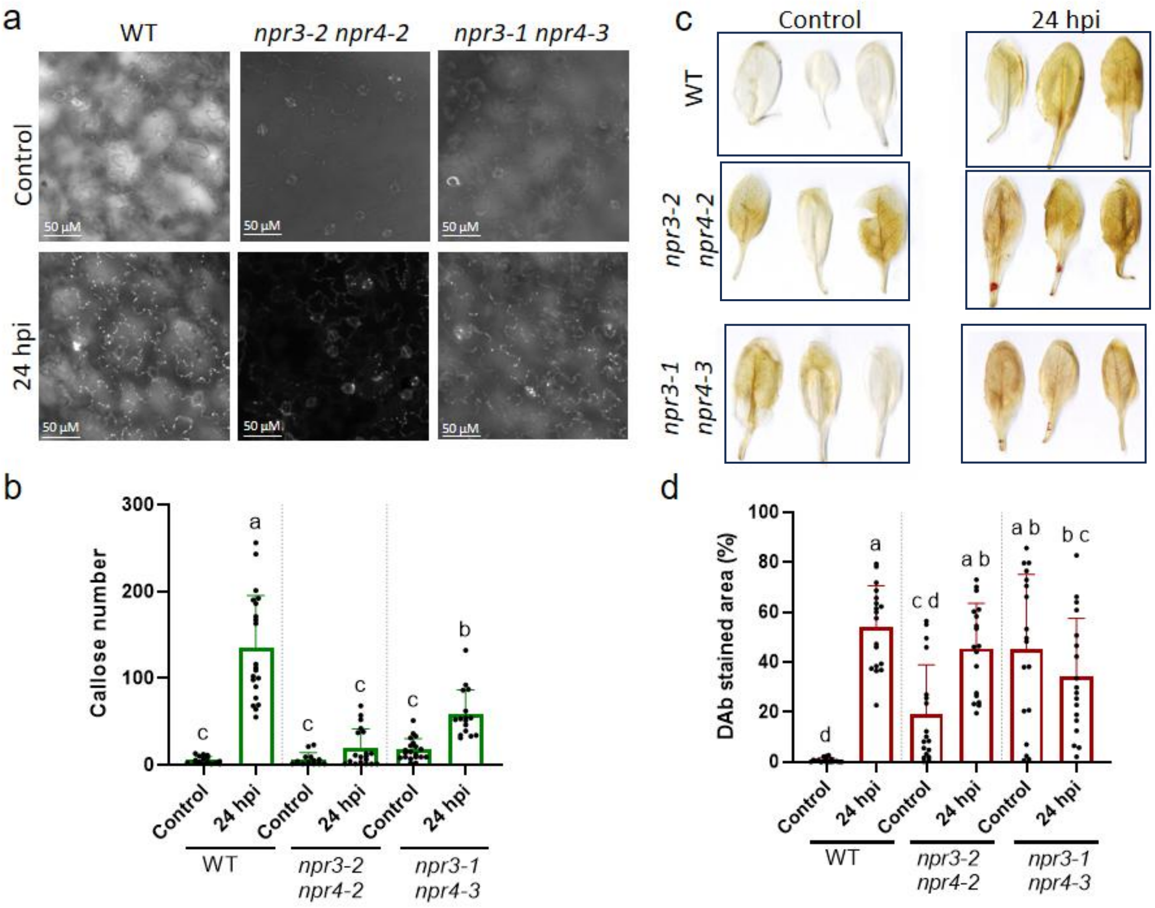

Supplementary Figure S6: *Psm*-induced callose and ROS accumulation in *Arabidopsis npr3 npr4* plants

a, Callose deposition (white dots) revealed by aniline blue staining in *Arabidopsis* wild-type (WT), *npr3-2 npr4-2*, and *npr3-1 npr4-3* leaves infected with *Psm* at 24 hpi. Representative images are shown. Control: healthy uninfected *Arabidopsis* leaves.

b, Numbers of callose depositions in the control and the *Arabidopsis* wild-type, *npr3-2 npr4-2*, and *npr3-1 npr4-3* leaves infected with *Psm* at 24 hpi. Bars represent 'means ± standard deviation (SD)' (n = 13-21). Data from three independent experiments were combined. Different letters above the bars denote significant differences ($p < 0.05$; one-way ANOVA with Tukey's test).

c, ROS accumulation (brown precipitates) revealed by DAB staining in *Arabidopsis* wild-type, *npr3-2 npr4-2*, and *npr3-1 npr4-3* leaves infected with *Psm* at 24 hpi. Representative images are shown. Control: uninoculated *Arabidopsis* leaves. (images in box photographed at the same time) (d) Percentages of leaf areas stained with DAB in the control and the *Arabidopsis* wild-type, *npr3-2 npr4-2*, and *npr3-1 npr4-3* leaves infected with *Psm* at 24 hpi. Bars represent 'means ± SD' ($n = 18$). Data from three independent experiments were combined. Different letters above the bars denote significant differences ($p < 0.05$; one-way ANOVA with Tukey's test).
